# The Role of Forgiveness Between Dysfunctional Thoughts and Anxiety in Older Adults’ Family Caregivers

**DOI:** 10.3390/geriatrics11010009

**Published:** 2026-01-08

**Authors:** Javier López, Maria Dolores Ortiz, Cristina Noriega

**Affiliations:** 1 Departamento de Psicología y Pedagogía, Facultad de Medicina, Universidad San Pablo-CEU, CEU Universities, 28925 Madrid, Spain; cristina.noriegagarcia@ceu.es; 2 Servicio de Psicología Clínica, Hospital Universitario Infanta Elena, Av. de los Reyes Católicos, 21, Valdemoro, 28342 Madrid, Spain; mdortizmunoz.1968@gmail.com

**Keywords:** forgiveness, avoidance, benevolence, caregiving, revenge, interpersonal relationships

## Abstract

Background/Objectives: Current studies have shown that caregiving anxiety is associated with an individual’s dysfunctional thoughts. The aim of this study was to assess the mediating effect of caregivers’ forgiveness (benevolence, lack of avoidance and lack of revenge) on the relationship between dysfunctional thoughts and anxiety in the informal caregivers of dependent older adults. Methods: Participants were 222 family caregivers. We conducted path analysis to test the hypothesized model. Results: We found a model that showed a good fit (χ^2^ = 3.410; χ^2^/gL = 5; *p* = 0.63; GFI = 0.994; CFI = 0.999; RMSEA = 0.001). It showed a direct and negative association between dysfunctional thoughts and lack of revenge, and this variable was related positively with both benevolence and lack of avoidance. In turn, benevolence was associated with lower levels of anxiety. The associations between dysfunctional thoughts and anxiety were mediated by caregiver forgiveness. Conclusions: Our research suggests the importance of health workers seeking to understand how individuals judge their avoidance, revenge and lack of benevolence, which affect individuals’ anxiety, for change. This study demonstrates the relevance of forgiving strategies in developing and testing informal caregiving assessments. It is necessary to detect and reduce avoidance and revenge related to caregivers. It is also necessary to detect and improve benevolence.

## 1. Introduction

Caregiving is a burdensome experience, and many family caregivers need psychological support [1]. Family caregiving of older adults has been related to chronic stress, which can affect individuals in the form of physical and psychological negative symptoms [2,3,4]. Many previous research studies have been conducted to identify factors associated with caregivers’ burden and depression. Nevertheless, family caregivers’ anxiety has been less studied. Previous results showed higher levels of anxiety in caregivers than in the general population. Female caregivers showed higher rates of anxiety in some research studies than male [5,6]. The research also suggests that dysfunctional beliefs about caregiving predict a caregiver’s anxiety and other distress-related variables [7,8], more so than symptoms of cognitive decline [9].

Dysfunctional beliefs about caregiving include statements that specify duties and obligations a caregiver must perform to be a “good caregiver”. These rigid, unrealistic or highly demanding statements may function as rules governing what caregivers should do, and they are prevalent among family caregivers [7]. Some dysfunctional beliefs about caregiving, such as the perception of sole responsibility and perfectionism, may invoke more distress among caregivers [10]. Dysfunctional thoughts are not specific to depression [7] and they are connected with cultural values [11]. These dysfunctional beliefs are common among caregivers and may lead to feelings of anxiety, besides being a burden [3].

The family caregiver and care recipient relationship is related to positive and negative emotions. As much as the caregiver may derive positive aspects from the relationship [12], they are many caregivers who experience anxiety and depression by the caregiving challenges [2,4,5]. Research on family relationships has found that forgiveness is often used to deal with anger when one relative is mad, upset or hurt by another relative, and that there is frustration, transgression or damage in returning the relationship to a normal stage when there is a high-quality, mutually beneficial close relationship. Forgiveness implies a motivational change to positive cognitions, emotions and behaviors from negative ones, and affects relationships. In this sense, forgiveness could be a mechanism through which family caregivers manage their dysfunctional thoughts by reducing avoidance and revenge [13], while simultaneously encouraging benevolent actions [14].

In the forgiveness process, some complex prosocial changes take place in certain basic interpersonal motivations. There are two negative affective states that correspond with two motivational systems that lead people’s responses to offense. The first is the feeling of being damaged by the attack, which corresponds to the need to avoid contact with the offender (both personal and psychological). The second affective state is the feeling of justified indignation, which corresponds to a motivation to take revenge. In caregiving contexts, revenge should not be interpreted as a literal intent to harm, but rather as a psychological response to perceived injustice or emotional injury. This response may be directed toward the care recipient, the healthcare system or other family members. Furthermore, there could be a benevolence motivation, which characteristically decreases when someone offends, causes harm or insults. The interaction of these three variables comprises forgiveness. After an offense has been forgiven, avoidance and revenge motivations would be inactive and benevolence would be active [14].

The conceptual model of caregiving stress proposes that stress and coping theories are well supported by the literature in caregiver research [4,11]. Furthermore, recent models—such as that proposed by Wang et al. [15]—provide updated empirical support for these frameworks, highlighting contemporary understandings of stress dynamics and coping mechanisms in caregiving contexts. From this perspective, primary stressors may produce emotional distress. However, the levels of distress experienced by caregivers may vary by their cultural values, appraisal style and coping styles. It is theoretically plausible that a potential action mechanism of the positive association between caregivers’ dysfunctional thoughts and anxiety involves its impact on their levels of forgiveness. Since caregiving may go on for many years, damage from daily dysfunctional thoughts might accumulate and eventually result in high anxiety. On the other hand, forgiveness may mediate its impact.

The caregiving conceptual model [4,11,15] understands coping as those efforts made by an individual to manage problems and emotions that generate discomfort, and that can influence the occurrence and intensity of the physical and psychological consequences associated with stress. More specifically, they are cognitions and behaviors that serve to evaluate the meaning of stressors, control or reduce stressful circumstances and modulate the affective activation that usually accompanies stress. Thus, in the case of caregivers, coping resources include several cognitive, affective and behavioral responses that help the person to manage their emotions, to face or ameliorate difficulties that arise on a daily basis and to maintain the psychological resilience and fortitude necessary to remain in the caregiving role for an extended period of time.

Forgiveness is a coping strategy which correlates with health [16]. Forgiveness can be considered one of these types of coping that mediates the impact of a stress-generating variable in caregivers, such as dysfunctional thoughts about caregiving. In the present study, forgiveness is conceptualized as interpersonal forgiveness, referring to the reduction in negative motivations (e.g., avoidance and revenge) and the increase in benevolent motivations toward a transgressor. Therefore, the focus of this study is on how caregivers direct forgiveness toward others, rather than on their capacity for self-forgiveness.

Despite the potential role that caregivers’ forgiveness can play in decreasing their anxiety, it has hardly received any meaningful research support. The main question of this research is as follows: when having to confront or restructure dysfunctional thoughts, does forgiveness, or its absence, help to explain caregivers’ anxiety? Our model hypothesizes that forgiveness may mediate the relationship between dysfunctional thoughts and anxiety. This reflects our hypothesis that more severe dysfunctional thoughts would make the lack of revenge more difficult. At the same time, the lack of avoidance and presence of benevolence may mediate the relationship between the lack of revenge and anxiety.

## 2. Materials and Methods

### 2.1. Participants

In this study, 222 family caregivers of dependent older adults dwelling in the community in several urban and rural locations of Spain participated. They were caring in the community for 61.29 h per week (SD = 64.04) and 6.71 years (SD = 6.81) on average. The caregivers’ average age was 54.78 (SD = 13.68), 63.1% were married, 69.8% were women, 37.3% were full-time workers and 22.7% were retired. They were caring for 1.04 people (SD = 0.43) on average. The average age of the older adults they were caring for was 80.16 (SD = 14.95), and 66.7% were women (see Table 1).

### 2.2. Procedure

The sample was obtained through snowball sampling from different older adults’ associations and organizations linked to the area of the older adults (i.e., Democratic Union of Pensioners and Retirees of Spain, Pandora Foundation). The highest percentage of caregivers surveyed were recruited from different autonomous communities of Spain.

Participants received information regarding the objectives of the study. The confidentiality and anonymity of the data were explained. They gave written informed consent and then received a self-applied survey that took 20 min approximately to complete.

### 2.3. Measures

This study included the following variables: caregivers’ gender (women and men), age, civil status (married, divorced, single or widowed), working status (working full-time, working part-time, retired, student, housewife–househusband, unemployed), socioeconomic status (medium-high, medium, or medium-low), number of years providing care, average daily hours providing care and number of older adults they were caring for, as well as the cared-for person’s age, sex and illness.

Dysfunctional thoughts were measured using the Dysfunctional Thoughts about Caregiving Questionnaire [10]. This 16-item scale evaluates the caregivers’ perception of sole responsibility and perfectionism (e.g., “It is logical for caregivers to give up their own needs, setting aside their own life satisfaction in favor of their relative’s needs”). The scale was designed to evaluate beliefs that may lead to a maladaptive coping in family caregivers. Answers range from 0 = “totally disagree” to 4 = “totally agree”. In the present study, the internal consistency (Cronbach’s alpha) was 0.85.

Forgiveness was assessed through the Spanish version of the Transgression-Related Interpersonal Motivations Inventory [13]. This scale includes 18 Likert-type items that measure possible responses to interpersonal offenses. The inventory has three subscales, namely, avoidance (7-item) (e.g., “I am trying to keep as much distance between us as possible”), revenge (5-item) (e.g., “I want to see him/her hurt and miserable”) and benevolence (6-item) (i.e., “Even though his/her actions hurt me, I have goodwill for him/her”). The answers range from 1 = “strongly disagree” to 5 = “strongly agree”. The avoidance and revenge subscales had to be reverse-coded. The inventory showed adequate internal consistency for avoidance (Cronbach’s alpha = 0.89), revenge (Cronbach’s alpha = 0.74) and benevolence (Cronbach’s alpha = 0.78) subscales.

Anxiety was assessed using the Hospital Anxiety Scale [17]. This scale includes seven Likert-type items to assess anxiety levels (i.e., “I feel tense or wound up”), with answers ranging from 0 to 3. In a review of 747 studies, it is found that, despite the word hospital that appears in its name, the scale can also be used in the general population [18]. In this study, the scale presents an internal consistency (Cronbach’s alpha) of 0.82.

### 2.4. Statistical Analysis

First, descriptive analysis and correlations between the outcome variables (dysfunctional thoughts, forgiveness and anxiety) were performed. We also developed Student *t*-tests (caregivers’ and care recipients’ sex), one-factor ANOVAs (civil status, working status, socioeconomic status, and cared-for person’s illness) and Pearson correlations (caregivers’ and care recipients’ age, years providing care, hours of care per week and number of people cared for) to analyze mean differences on the outcome variables by sociodemographics. Path analysis was developed to evaluate the relationship between participants’ dysfunctional thoughts (considered as a vulnerability variable) and their anxiety. We also tested if this relationship was mediated by the forgiveness subscales considered in this study as protective variables (benevolence, lack of revenge and lack of avoidance). The maximum-likelihood method was used to estimate the significance of all model path coefficients and fit statistics.

## 3. Results

Lack of revenge correlated negatively with dysfunctional thoughts, and positively with benevolence and lack of avoidance. Also, benevolence and lack of avoidance correlated positively (descriptives and correlations are shown in Table 2).

We then analyzed differences in dysfunctional thoughts, anxiety and forgiveness by sociodemographic characteristics. We found that older caregivers showed more dysfunctional thoughts (r = 0.147; *p* < 0.038) and caregivers caring for more years showed less revenge (r = 0.168; *p* < 0.045). There were also differences by sex (t = 2.186; *p* = 0.030), showing women (M = 8.47; SD = 4.36) having higher levels of anxiety than men (M = 7.32; *p* = 3.86). In contrast, no differences were found in the outcome variables by caregivers’ civil, working and socioeconomic status, average daily hours providing care, number of older adults they were caring for and care recipients’ age, sex and illness.

**Figure 1:**
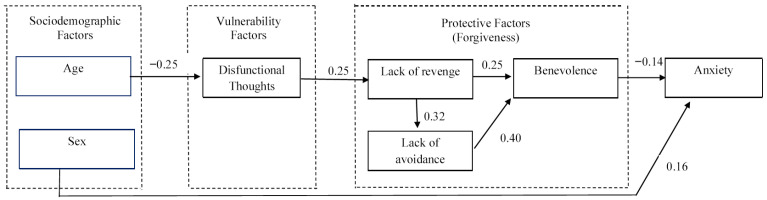
Final structural equation model with standardized regression weights.

We found a model that showed a good fit (χ^2^ = 8.305; χ^2^/gL =14; *p* = 0.87; GFI = 0.990; CFI = 0.999; RMSEA = 0.001) [19,20]. This model showed a direct and negative association of dysfunctional thoughts with lack of revenge, and this variable was related positively with both benevolence and lack of avoidance. In turn, lack of avoidance related positively with benevolence. Finally, benevolence was related to lower levels of anxiety (see Figure 1, Table 3 and Table 4). The model explained 24.7% of the variance.

## 4. Discussion

The hypothesized model was generally supported by the results found. Dysfunctional thoughts were associated with lower levels of forgiveness, although such relationships were observed directly in the domain of lack of revenge only. Caregiver benevolence mediated the association between the lack of revenge and anxiety. Lack of revenge was directly related with benevolence and indirectly related with benevolence through lack of avoidance. Overall, the results support the importance of increasing the benevolence and lack of avoidance by caregivers and one way of doing so may be to become less revengeful against care recipient offenses.

The results showed that only one forgiveness domain—lack of revenge—is significant and negatively associated with dysfunctional thoughts about caregiving. Coincidentally, this domain (lack of revenge) had been shown to invoke more dispositional empathy (ability to experience others’ emotions) compared to other forgiveness domains such as benevolence and lack of avoidance [21]. These results may explain why these dysfunctional thoughts about caregiving lead to more anxiety in caregivers. The mediating effect of forgiveness seems to support the hypothesis that more intense dysfunctional thoughts about caregiving lead to more anxiety, partly because they reduce the level of forgiveness in caregivers. It is reasonable to think that dysfunctional thoughts about caregiving might debilitate forgiveness by anxious caregivers. A perfectionist caregiver may have difficulty asking for forgiveness and avoiding retaliation. Furthermore, caregivers’ lack of revenge could promote a lack of avoidance and benevolence. Nevertheless, further studies should be developed to determine potential variables which may mediate the effect of dysfunctional thoughts about caregiving on forgiveness.

The effect of the lack of revenge on caregivers’ anxiety was mediated by benevolence and lack of avoidance. The previous literature has found that forgiveness predicts less caregiver anxiety [22,23]. Furthermore, based on this result, some mechanisms are postulated through which anxiety is reduced by forgiveness. First, forgiveness is a partly cognitive process that provides a different perspective on events [24,25]. Individuals capable of forgiving have fewer anxiety-provoking thoughts. Second, there is an increase in forgiveness-strengthening feelings such as compassion, generosity and even love [24]. As a result, forgiving neutralizes negative emotions and can positively impact anxiety. Third, the likelihood of anxious symptomatology is reduced due to the fact that forgiveness strengthens benevolent actions and concerns for well-being [24].

As a caregiver cultivates and practices the cognitive, emotional and behavioral issues implied in the forgiveness process, the person is gaining personal strength, which should be a buffer against dysfunctional thoughts. Although a previous study hypothesized that dysfunctional thoughts about caregiving may reflect religious (Catholicism-related) values and norms (e.g., self-sacrifice), which are quite present in Latin societies [26], we consider that one aspect of a caregiver’s life that might buffer anxiety is his/her religious values [22]. In addition, researchers often find that one’s religious values and norms are related to the level of forgiveness, as a previous meta-analysis has found [27]. The negative emotions, cognitions and behaviors related to a lack of forgiveness are replaced by neutral or more positive ones when the person is able to forgive. This substitution can take place in one transformative experience or step by step over time [22]. Forgiveness is a critical part of many religious values, and it should be distinguished from excusing and forgetting. Forgiveness has been used since antiquity in religious communities and it is considered a key domain of religiousness, which is essential for studies where some measure of health serves as an outcome [28]. Given this, it may be beneficial for professionals working with family caregivers to be aware of the potential influence of religious values [15]. When appropriate and with sensitivity to individual preferences, practitioners might consider exploring caregivers’ religious or spiritual frameworks as part of a holistic assessment. This could help tailor interventions that resonate with caregivers’ belief systems, potentially enhancing their emotional resilience and capacity for forgiveness. Future research should further examine the role of religion in caregiving contexts to inform culturally and spiritually sensitive practices.

The anxiety of female caregivers is higher than that of male caregivers. As some previous research has reported, family caregiving has a greater impact on women’s anxiety. Female caregivers are more negatively affected than men, namely, women are somewhat more strained [5,6,29]. The observed gender differences in anxiety among caregivers may be partially explained by the presence of dysfunctional thoughts related to caregiving. In our study, female caregivers reported significantly higher levels of dysfunctional thoughts compared to their male counterparts. Maladaptive cognitions such as excessive responsibility, guilt and self-imposed caregiving standards may contribute to the heightened anxiety experienced by women. This interpretation aligns with previous findings suggesting that women are more likely to internalize caregiving roles and experience greater emotional impact. Therefore, dysfunctional thoughts may serve as a cognitive mechanism underlying the gender disparity in caregiver anxiety, highlighting the importance of addressing these beliefs in interventions aimed at reducing caregiver anxiety. Nevertheless, more research is needed to identify the relevant dimensions that distinguish male and female caregivers’ anxiety.

This study shows that dysfunctional thoughts are significantly higher in older caregivers. A previous study [26] showed that middle-aged caregivers (under 60) showed less dysfunctional thoughts about caregiving than older adult caregivers (60 years and over). Furthermore, the validation study of the Dysfunctional Thoughts about Caregiving Questionnaire found that dysfunctional thoughts are significantly correlated with caregivers’ age [10]. Probably, the perception of sole responsibility and perfectionism are more prevalent in older caregivers.

Finally, despite the fact that we hypothesized a significant positive correlation between dysfunctional thoughts and anxiety measures, the correlation was non-significant. The fact that caregivers with dysfunctional thoughts about caregiving do not show more anxiety can be explained by the common belief in caregivers that affirms that good caregivers should cope with their caregiving emotions by themselves. Other caregivers who strongly believe that good caregivers must not complain or share any negative feeling can feel guilty if they do so (e.g., anxiety), because this behavior is inconsistent with the rule that a good caregiver should not complain or express any negative feelings. Some items of the Dysfunctional Thoughts about Caregiving Questionnaire reflect these ideas vividly (e.g., “If a caregiver has feelings of embarrassment and rejection toward his/her relative, it is because the caregiver is failing in some way with his/her caregiving duties” item number 10; “Good caregivers should remain happy and in good spirits all day long to deal adequately with the daily tasks of caregiving” item number 11; “A good caregiver should never get mad or lose control with the person that is being cared for” item number 12).

There are several limitations in this study. First, the cross-sectional design of this study does not allow for conclusions on causality. Second, participants were mainly women and they were collected by convenience sampling. Future studies should include more diverse samples with a higher percentage of men to be able to generalize the results. Nevertheless, more of the caregivers are women. Third, due to the scope of the study, the potential impact factors on forgiveness, such as religion or empathy, were not examined in the study.

## 5. Conclusions

Despite the above limitations, these results support the hypothesis that forgiveness mediates the impact of dysfunctional thoughts about caregiving on anxiety. The relationship between forgiveness and anxiety is important for gerontologists to consider [24]. The results of this study suggest that considering unforgiveness may be particularly relevant when clinicians attend to older adults’ family caregivers who present with anxiety and dysfunctional thoughts about caregiving. Based on the findings of this study, we recommend that professionals supporting family caregivers consider interventions that specifically target key components of interpersonal forgiveness. In particular, reducing revenge motivations may be especially beneficial, as this dimension was most strongly associated with dysfunctional thoughts. This approach is consistent with Enright’s forgiveness triad, which emphasizes the therapeutic value of forgiving others, receiving forgiveness and self-forgiveness [24]. Although our study focused on interpersonal forgiveness, future interventions could also explore the role of self-forgiveness where relevant. Furthermore, recent models—such as that proposed by Wang et al. [15]—provide updated empirical support for these frameworks, highlighting contemporary understandings of stress dynamics and coping mechanisms in caregiving contexts. In this regard, we suggest that healthcare professionals consider using the Dysfunctional Thoughts about Caregiving Questionnaire (DTCQ) as a tool to better understand caregivers’ cognitive patterns. Identifying and addressing dysfunctional thoughts may help reduce anxiety and improve emotional well-being.

To enhance the clinical applicability of these findings, we also propose the integration of structured interventions such as forgiveness training programs, compassion-focused therapies and cognitive-behavioral approaches tailored to caregivers. These strategies may foster emotional regulation, empathy and adaptive coping, ultimately contributing to improved mental health outcomes. As highlighted by López et al. [25], forgiveness-based interventions have shown promise in older adult populations, and adapting these to address caregiving-specific cognitions may further enhance their effectiveness.

## Figures and Tables

**Table 1:** Demographic characteristics and caregiving-related information of the participants (N = 222).

	Total (n = 222)	Women (n = 154)	Men (n = 68)
Civil Status			
Married	63.1% (140)	61.4% (94)	66.2% (45)
Divorced	10.8% (24)	12.4% (19)	7.4% (5)
Single	20.7% (46)	19.0% (29)	25.0% (17)
Widowed	5.4% (12)	7.2% (11)	1.5% (1)
Age	54.78 (13.68)	55.15 (13.45)	53.95 (14.41)
Working Status			
Working full time	37.3% (82)	31.4% (48)	50.0% (33)
Working part time	10.5% (23)	10.5% (16)	10.6% (7)
Retired	22.7% (50)	20.9% (32)	27.3% (18)
Student	3.6% (8)	3.9% (6)	3.0% (2)
Housewife/-husband	17.3% (38)	24.2% (37)	1.5% (1)
Unemployed	8.6% (19)	9.2% (14)	7.6% (5)
Socioeconomic Status			
Medium-high	3.2% (7)	3.3% (5)	3.0% (2)
Medium	57.7% (127)	55.9% (85)	61.2% (41)
Medium-low	39.1% (86)	40.8% (62)	35.8% (24)
Years Providing care	6.71 (6.81)	6.18 (5.06)	7.64 (9.06)
Hours of care per week	61.29 (64.04)	71.26 (66.20)	45.54 (57.82)
Care recipient’s age	80.16 (14.95)	80.03 (15.34)	80.25 (14.48)
Care recipient’s sex			
Men	24.7% (37)	24.5% (23)	25.5% (14)
Women	66.7% (100)	68.1% (64)	65.5% (36)
Both	6.7% (10)	5.3% (5)	7.3% (4)
Number of people cared	1.04 (0.43)	1.00 (0.4)	1.09 (0.39)
Cared person illness			
Dementia	59.2% (132)	61% (94)	44.1% (30)
Non-dementia	40.8% (91)	39.0% (60)	55.9% (38)

**Table 2:** Pearson correlations, means, and standard deviations among dysfunctional thoughts, anxiety and forgiveness dimensions.

	Dysfunctional Thoughts	Anxiety	Benevolence	Lack of Revenge	Lack of Avoidance
Dysfunctional Thoughts					
Anxiety	0.244				
Benevolence	−0.024	−0.130 *			
Lack of revenge	−0.253 ***	−0.099	371 ***		
Lack of avoidance	−0.041	−0.108 ***	0.475 **	0.319 ***	
Mean	52.00	8.01	20.51	21.66	20.64
SD	15.94	3.60	5.53	3.58	7.02

*** *p* < 0.001; ** *p* < 0.01; * *p* < 0.05.

**Table 3:** Final structural equation model results.

			RW	SRW	SE	CR	*p*
Age	→	Dysfunctional thoughts	0.105	0.131	0.053	1.965	0.049
Dysfunctional thoughts	→	Lack of revenge	−0.087	−0.253	0.022	−3.883	0.001
Lack of revenge	→	Lack of avoidance	0.626	0.319	0.125	4.987	0.001
Lack of revenge	→	Benevolence	0.379	0.245	0.093	4.058	0.001
Lack of avoidance	→	benevolence	0.312	0.397	0.048	6.571	0.001
Benevolence	→	Anxiety	−0.090	−0.137	0.043	−2.083	0.037
Sex	→	Anxiety	1.210	0.158	0.504	2.400	0.016

RW = regression weights; SRW = standardized regression weights; SE = standard error; CR = critical ratio.

**Table 4:** Direct and indirect effects using bootstrapping.

			Direct Effect	*p*	Indirect Effect	*p*
Age	→	Dysfunctional thoughts	0.131	0.045	-	-
Dysfunctional thoughts	→	Lack of revenge	−0.253	0.004	-	-
Lack of revenge	→	Lack of avoidance	0.319	0.003	-	-
Lack of revenge	→	Benevolence	0.245	0.005	0.126	0.003
Lack of avoidance	→	Benevolence	0.397	0.004	-	-
Benevolence	→	Anxiety	−0.137	0.078	-	-
Sex	→	Lack of revenge	0.158	0.015	-	
Age	→	Lack of revenge	-	-	−0.033	0.027
Age	→	Lack of avoidance	-	-	−0.011	0.017
Age	→	Benevolence	-	-	−0.012	0.020
Age	→	Anxiety	-	-	0.002	0.050
Dysfunctional thoughts	→	Lack of avoidance	-	-	−0.081	0.003
Dysfunctional thoughts	→	Benevolence	-	-	−0.094	0.003
Dysfunctional thoughts	→	Anxiety	-	-	−013	0.042
Lack of revenge	→	Anxiety	-	-	−0.05	0.071
Lack of revenge	→	Anxiety	-	-	−0.05	0.071

## Data Availability

The data that support the findings of this study are openly available in the Open Science Framework repository accessed on 7 June 2023 at https://osf.io/m84ps/.

## Abbreviations

The following abbreviations are used in this manuscript:

M	Mean
SD	Standard Deviation
χ^2^	Chi-Square
χ^2^/gL	Chi-Square per Degrees of Freedom
*p*	*p*-value
GFI	Goodness of Fit Index
CFI	Comparative Fit Index
RMSEA	Root Mean Square Error of Approximation
RW	Regression Weights
SRW	Standardized Regression Weights
SE	Standard Error
CR	Critical Ratio
